# Editorial: Dystonia and tremor

**DOI:** 10.3389/dyst.2025.14589

**Published:** 2025-05-21

**Authors:** Pattamon Panyakaew, Aparna Wagle Shukla

**Affiliations:** 1 Division of Neurology, Department of Medicine, Faculty of Medicine, Chulalongkorn Excellence Centre on Parkinson’s Disease and Related Disorders, King Chulalongkorn Memorial Hospital, Chulalongkorn University, Bangkok, Thailand; 2 Norman Fixel Institute for Neurological Diseases, College of Medicine, University of Florida, Gainesville, FL, United States

**Keywords:** dystonia, tremor, dystonic tremor, task-specific tremor, dystonic tremor syndrome

The definition of “Dystonia and tremor” is heterogeneous and has been recently revisited in 2024 by an expert panel of the MDS Dystonia Study Group and the Dystonia Coalition. It has now been explicitly redefined to refer only to rhythmic oscillatory movements in dystonia. In contrast, the arrhythmic jerky movements seen in dystonia, which were also previously classified as dystonia and tremor, should now be renamed as jerky repetitive dystonia and not be included in the dystonia and tremor category [1–3]. This may help to create more standardized terminology in research and clinical practice. The studies in this special issue of *Dystonia and Tremor* provide updated insights into dystonia and tremor, particularly in isolated focal dystonia, with detailed characterization of the clinical features and physiological aspects of isolated cervical dystonia (CD) and task-specific dystonia (TSD) presenting with tremor. This issue also advances the understanding of gait and balance impairments in CD patients with tremor, and nonmotor symptoms in children and adults with dystonia underscoring the importance of multidisciplinary care for this patient population.

Beylergil et al. determined the prevalence of tremor in CD patients enrolled in the Dystonia Coalition cohort. The study found that approximately 45% of patients, particularly women, experienced head tremor, with nearly 75% exhibiting irregular head tremor (better classified as jerky dystonia) and 25% presenting with regular head tremor. Predictors of head tremors included increased disease severity, increased disease duration, and increased age, in this order, whereas the presence of regular head tremor was associated with decreased disease severity and older age. This underpins that jerky dystonia and regular head tremor seen in CD should be regarded as distinct entities.

Jabarkheel and Wagle Shukla prospectively compared the electrophysiologic characteristics of head and arm tremor in patients with focal CD vs segmental dystonia. While the mean frequency of the head tremor was observed to be low (4.3 ± 0.9; range 3.5–6 Hz), the arm tremor had a slightly higher frequency (5.5 ± 0.6; range 3.5–7 Hz). The frequency of head tremor was higher in younger participants than in older participants, as previously described in patients with essential tremor (ET). When comparing focal vs. segmental dystonia, the head tremor in CD had a lower peak frequency and amplitude with a longer EMG burst duration. Arm tremor in patients grouped as focal dystonia (CD plus arm tremor without dystonic features) had a lower amplitude compared to segmental dystonia (CD plus arm tremor with dystonic features). Head and arm tremor tended to be less severe in patients who reported alcohol responsiveness. The study concluded that the physiological characteristics of tremor in focal and segmental dystonia differ to some extent, indicating that the progression of dystonia symptoms across body regions may influence the underlying physiology of co-occurring tremor.

The effects of botulinum toxin (BoNT) injection on the regularity of head oscillation in CD were investigated in a small sample size (N = 8 with documented head movements by the magnetic search coil) Agharazi et al. The regularity of head tremor was quantified by calculating the dispersion of head movements in time series values. BoNT injection could change the regularity of head tremor to a certain “set-point” in the oscillatory network possibly by modulating proprioceptive feedback to the head neural integrator [4]. In addition, the randomness of head movements was not changed by BoNT injection, supporting that the head movements in this study were consistent with jerky dystonia rather than tremor based on the current viewpoint. Overall, the amplitude and frequency of head movements decreased with BoNT injection, with a pronounced reduction in head orientation in patients with high-intensity head oscillation prior to the injection.

Whether task-specific tremor (TST) should be classified as a form of task-specific dystonia (TSD) or a variant of ET remains unclear. The electrophysiology of TST is poorly characterized. Kuo and Chen reviewed the current evidence on the underlying physiology of TST. The majority of the studies were conducted in patients diagnosed with primary writing tremor and TST presenting in musicians. Electromyographic results showed co-activation between the antagonist pairs and overflow muscle activities to the adjacent muscles, similar to dystonia. However, the loss of inhibition at the spinal, brainstem, and cortical levels was not identical to dystonia. Reciprocal inhibition, the physiological technique to assess spinal inhibition, was normal in TST. GABAergic cortical inhibition was slightly impaired, while the cortical silent period was within the normal range. Functional imaging revealed reduced functional connectivity between the cerebellum and other parts of the brain, but less widespread compared to dystonia. Taken together, TST may be a subtype of dystonia and tremor rather than ET. Nevertheless, it may be a separate entity since it is not entirely congruent with the physiology of dystonia and tremor.

Zhou et al. compared the prevalence of non-motor features such as depression, anxiety, fatigue, and sleep disturbances in pediatric-onset versus adult-onset dystonia. As expected, pediatric-onset dystonia was more commonly associated with the generalized form, whereas adult-onset dystonia tended to present as focal dystonia. Interestingly, aside from a lower rate of sleep disturbances in children, the prevalence of fatigue, anxiety, and depression was comparable between pediatric and adult patients with dystonia.

Finally, gait and balance problems have been identified in patients with CD. However, these aspects have never been addressed in CD with head tremor. Wagle Shukla et al. investigated the clinical and spatiotemporal parameters of gait in this specific group. They demonstrated that nearly half of the patients with CD and tremor experienced clinical gait and balance difficulties, including slower walking speed and impaired performance on the Berg Balance Scale. In their assessments, more than 20% of patients had a shorter step length, wider stride width, and increased double support time when walking on a gait mat compared to healthy individuals, suggesting that an abnormal cerebellar network contributes to these findings. However, when compared with ET and patients with orthostatic tremor, the dystonia and tremor group exhibited less pronounced abnormalities in objective gait and balance variables, suggesting that a relatively lower degree of dysfunction within the cerebellar network was present. The study also highlighted that gait and balance dysfunction in CD with head tremor may also stem from factors beyond cerebellar dysfunction, including impairments in vestibular and proprioceptive pathways due to abnormal head positioning and constant head shaking. Reduced control of voluntary neck movements may further hinder navigation in complex environments. These findings emphasize the importance of incorporating rehabilitation strategies into outpatient management plans for dystonia and tremor.

In summary, this Special Issue of *Dystonia and Tremor* emphasizes the clinical characteristics, physiological aspects, and pathophysiological understanding of dystonia and tremor. The repetitive jerky movements of a specific body part in dystonia should no longer be classified as a tremor. Physiological findings can offer clinicians valuable insights to improve diagnosis and patient management while also guiding researchers in designing more robust studies. The implementation of more precise definitions for dystonia and tremor represents an essential advance in generating more homogenous evidence in this field.
